# The association between physical activity and vertebral dimension change in early adulthood – The Northern Finland Birth Cohort 1986 study

**DOI:** 10.1016/j.bonr.2021.101060

**Published:** 2021-04-09

**Authors:** Elsi Autio, Petteri Oura, Jaro Karppinen, Markus Paananen, Juho-Antti Junno, Jaakko Niinimäki

**Affiliations:** a Research Unit of Medical Imaging, Physics and Technology, Faculty of Medicine, University of Oulu, Oulu, Finland; b Center for Life Course Health Research, Faculty of Medicine, University of Oulu, Oulu, Finland; c Medical Research Center Oulu, Oulu University Hospital and University of Oulu, Oulu, Finland; d Finnish Institute of Occupational Health, Oulu, Finland; e Department of Radiology, Oulu University Hospital, Oulu, Finland; f Cancer and Translational Medicine Research Unit, Faculty of Medicine, University of Oulu, Oulu, Finland; g Department of Anatomy, Faculty of Medicine, University of Oulu, Oulu, Finland; h Department of Archaeology, Faculty of Humanities, University of Oulu, Oulu, Finland

**Keywords:** Lumbar vertebrae, Magnetic resonance imaging, Osteoporosis, Physical activity, Spine

## Abstract

Small vertebral size is a well-known risk factor for vertebral fractures. To help understanding the factors behind vertebral size, we aimed to investigate whether physical activity and participation in high-impact exercise are associated with the growth rate of the vertebral cross-sectional area (CSA) among young adults. To conduct our study, we utilized the Northern Finland Birth Cohort 1986 as our study population (*n* = 375). Questionnaire data about physical activity was obtained at 16, 18 and 19 years of age and lumbar magnetic resonance imaging scans at two timepoints, 20 and 30 years of age. We used generalized estimating equation (GEE) models to conduct the analyses. We did not find any statistically significant associations between vertebral CSA, physical activity, and high-impact exercise in our study sample. We conclude that neither physical activity nor high-impact sports seem to influence the change in vertebral CSA among young adults.

## Highlights

• Physical activity does not influence the growth rate of the vertebral body.
• High-impact sports are not associated with the change in vertebral CSA among adults.
• The study was conducted using longitudinal MRI data.

## 1 Introduction

Osteoporosis is a risk factor of non-traumatic skeletal fractures (Ruyssen-Witrand et al., 2007). Globally 20% of men and 33% of women have augmented risk for osteoporotic fractures (International Osteoporosis Foundation, 2017). Vertebral fractures are among the most common osteoporotic fractures. Vertebral fracture risk is significantly influenced by bone geometry and vertebral bone mineral density (BMD) (Bouxsein and Karasik, 2006; Odvina et al., 1988). Smaller vertebral cross-sectional area (CSA) indicates an increased susceptibility to vertebral fractures (Ruyssen-Witrand et al., 2007).

Nutrition and exercise habits influence the BMD (Heaney et al., 2000). However, it is largely unknown, which lifetime factors are associated with the growth rate of vertebral dimensions. Other factors which influence the bone size and growth are calcium and vitamin D intake as well as genetics (Heaney et al., 2000).

There are several cross-sectional studies demonstrating age-related increase in vertebral dimensions among elderly adults in general and elderly males in particular (Mosekilde and Mosekilde, 1990; Seeman, 2001) However, the magnitude, timing and sex-relatedness of this increase is still controversial (Junno et al., 2015). It has been suggested that periosteal apposition results in increased CSA of the vertebral corpus to compensate for the decline in BMD with age (Seeman, 2001). In our previous study, we demonstrated age-related increase in vertebral dimensions in young adulthood, between the ages of 20 and 30, among both sexes (Autio et al., 2019). These findings raise the question whether lifestyle factors such as physical activity (PA) and participation in high-impact sports may affect vertebral growth rate in young adulthood, and potentially lower the risk of vertebral fractures.

PA and especially exercise involving intense loading with high impact forces seem to have a positive influence on BMD (Heaney et al., 2000; Tenforde et al., 2018) and exercise has also beneficial effects on bone geometry (Tenforde and Fredericson, 2011). However, based on cross-sectional studies, these effects may not concern the vertebral CSA. In their study, Junno et al. (Junno et al., 2013; Junno et al., 2011) found no connection between the vertebral strength and PA among 558 individuals aged 21, neither between the level of PA and the vertebral CSA. Nikander et al. had similar results in their study (Nikander et al., 2010). Cöster et al. (Cöster et al., 2016) did not find any correlation between the level of PA and the risk of vertebral fractures. Interestingly, in a middle-aged sample, moderately larger vertebral CSA was detected among women who participated in high-impact sports at least once a week compared to women who did not exercise (Oura et al., 2017a). Among men, participation in high-impact exercise did not influence the CSA. In another study, a similar result was found also regarding lifetime leisure-time physical activity (LTPA) (Oura et al., 2016), as a high level of LTPA predicted larger CSA among women but not among men. However, the influence of PA in young adulthood on the vertebral CSA has not been evaluated before in longitudinal studies.

PA in young adulthood has been shown to predict PA in later adulthood. The level of PA at the age of 15 seems to predict the level of PA at the age of 30 (Engström, 1986). It has also been concluded that the results of physical tests and the level of PA at the age of 16 predicted accurately the level of PA in adulthood (Barnekow-Bergkvist et al., 1998).

The aim of this study was to evaluate the associations between physical activity level, participation in impact exercise, and vertebral CSA with our longitudinal magnetic resonance imaging (MRI) data from two time points 10 years apart. We hypothesized that the previously observed increase in CSA over the follow-up could be linked to the level of PA and participation in high-impact sports at baseline.

## 2 Material and methods

### 2.1 Study population

**Fig. 1:**
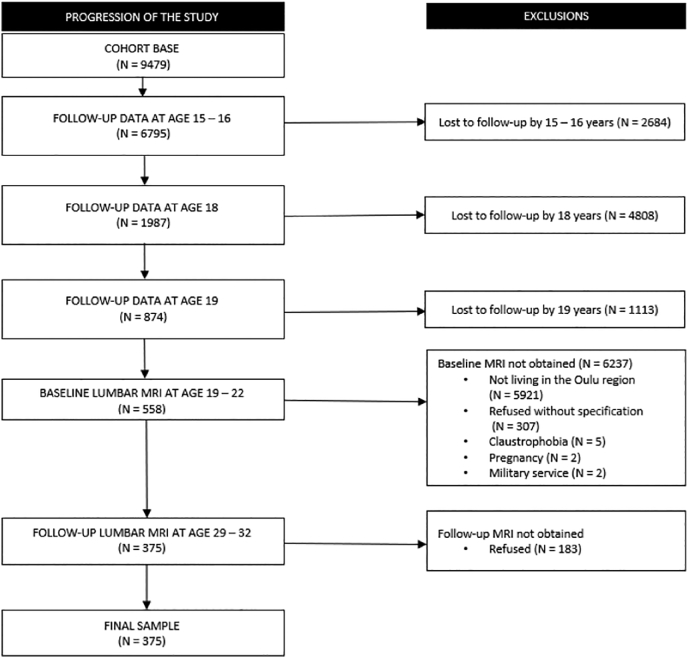
Progression of the study and reasons for exclusions.

The source population was a prospective, population-based birth cohort study, which initially covered 99% of children whose expected dates of birth fell between July 1, 1985 and June 30, 1986 in Northern Finland (Järvelin et al., 1993). This was equivalent to 9479 children. Our study population was a sub-cohort and consisted of those who 1) attended clinical examinations and filled in questionnaires about their health and lifestyle habits in adolescence and early adulthood, and 2) participated in repeated MR scans of the lumbar spine 10 years apart (Fig. 1).

At the age of 15–16, in 2001–2002, 9215 cohort members were invited to answer questionnaires and to participate in clinical examinations. 7182 adolescents (78% of those invited) responded to the questionnaires and 6795 adolescents (74% of those invited) participated in clinical examinations. At the age of 18, 2969 received a postal questionnaire (response rate 67%, *n* = 1987). At the age of 19, 874 individuals filled in the questionnaire and participated in the clinical examination (44% of those invited). At the age of 19–22, in 2005–2008, those who lived within a 100 km radius of the city of Oulu and had attended in the earlier follow-ups (*n* = 874) were invited to MRI. 558 individuals (64% of those invited) underwent the baseline MRI. Finally, at the age of 29–32, in 2015–2017, those who had undergone the baseline MRI, were invited to the follow-up MRI. 375 individuals (43% of those originally invited to baseline MRI) underwent the follow-up MRI. Our final sample size was therefore 375 (Autio et al., 2019; Paalanne, 2011; Paananen, 2011). A flow chart of the data collection and reasons for exclusions are represented in Fig. 1.

### 2.2 Magnetic resonance imaging of the lumbar spine

The baseline and the follow-up MR scans were taken by using 1.5-T imaging (repetition time 3960 ms, echo time 116 ms, echo train length 29, number of excitations 4, acquisition matrix 448 × 224 px, field of view 280 × 280 mm, slice thickness 4 mm, and interslice gap 1 mm) and T2-weighted scans. More detailed information is presented in our previous study (Autio et al., 2019).

### 2.3 Vertebral measurements

**Fig. 2:**
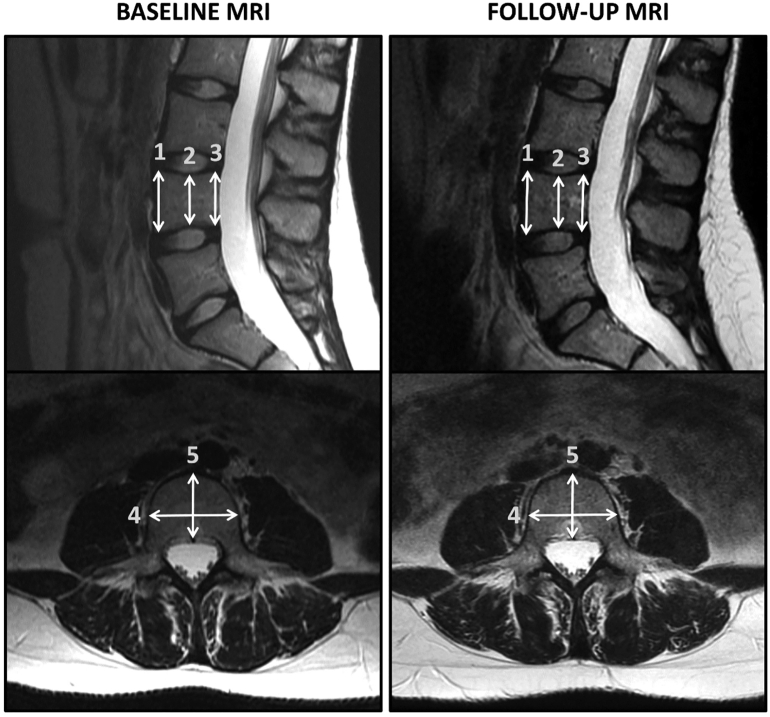
Annotated MR scans of the same individual at age 21 (Baseline MRI) and at age 30 (Follow-up MRI). Above: Midsagittal scans with anterior height (Ruyssen-Witrand et al., 2007), minimum height (International Osteoporosis Foundation, 2017) and posterior height (Bouxsein and Karasik, 2006) measurements marked on L4. Below: Midaxial scans of L4 with annotated width (Odvina et al., 1988) and depth (Heaney et al., 2000) measurements.

We chose to measure the dimensions of the fourth lumbar vertebra (L4). We were interested in L4 because it is subject to significant loading due to its caudal location and because there are many similar studies where researchers had investigated precisely L4 (Junno et al., 2015; Junno et al., 2013; Junno et al., 2011; Oura et al., 2017a; Oura et al., 2016; Bogduk, 2012; Oura et al., 2017b; Oura et al., 2017c; Junno et al., 2009). In addition, L4 is a good proxy for other lumbar vertebrae and their response to external factors (Oura et al., 2016; Brinckmann et al., 1989). In our previous study we have explained extensively how dimensions were chosen and how measures were calculated (Autio et al., 2019). In brief, we had data on vertebral height, width, depth and CSA. Sagittal and axial slices of L4 were utilized as we calculated the means for height, width and depth. To calculate the vertebral CSA we used ellipsoid formula CSA = π x a/2 x b/2, in which a = vertebral width (mean of measured widths) and b = vertebral depth (mean of measured depths) (Peel and Eastell, 1994; Tabensky et al., 1996). These dimensions are represented in a Fig. 2. MRI has been proven to be an accurate tool when investigating vertebral dimensions (Junno et al., 2009). In our previous study we have shown that our measurements were accurate and reliable (Autio et al., 2019).

### 2.4 Anthropometry and lifestyle habits

In 2001–2002, at the 15/16-year follow-up, questionnaire data and clinical data were received from 6795 adolescents. The questionnaire collected data on adolescents' smoking habits and PA, and participants' height and weight were measured by a trained study nurse. We also obtained similar questionnaire data with identically formulated questions from 18- and 19-year follow-ups and used the most recent data which was available.

Smoking habits were reported by answering to the question ‘Do you currently smoke?’. The response alternatives were 1) no, 2) occasionally, 3) on one day/week, 4) on 2–4 days/week, 5) on 5–6 days/week, 6) on 7 days/week (Autio et al., 2019). Those who chose alternative 1 were classed as ‘non-smokers’, those who chose alternatives 2–3 were classed as ‘occasionally-smokers’, and those who chose alternatives 4–6 were classed as ‘smokers’.

PA level was determined by the question ‘How often do you exercise outside school hours for a period of at least 20 minutes?’. The response alternatives were 1) never, 2) once/month or less often, 3) 2–3 times/month, 4) once/week, 5) 2 times/week, 6) 3 times/week, 7) 4–6 times/week, 8) daily (Autio et al., 2019). Those who responded exercising less than once a week (corresponding alternatives 1–3) were classed as ‘non-active’, those who responded exercising 1–3 times a week (corresponding alternatives 4–6) were classed as ‘semi-active’, and those who responded exercising at least 4 times a week (corresponding alternatives 7–8) were classed as ‘active’.

To observe the influence of high-impact sports on vertebral dimensions, we used the self-reported sports participation data. If an adolescent reported participating at least once a week in at least one of the following sports, the person was classed as ‘high-impact’. We considered the following sports as high-impact sports: running, soccer, ice hockey, floorball, rinkball, Finnish baseball, basketball, aerobics, volleyball, badminton and tennis (Oura et al., 2017a). Individuals who did not participate in any high-impact sport were classed as ‘low impact’. We further summed each individual's cumulative impact exercise as follows: If an individual participated in one impact sport at least once a week, they were given value 1. If they participated in two impact sports at least once a week, they were given value 2 and so on. Men had values between 0 and 8 and women had values between 0 and 5. We called the variable representing cumulative impact exercise as Summed Impact.

### 2.5 Statistical analysis

Statistical analyses were conducted by using SPSS (IBM, Armonk, NY, USA) version 25, 64-bit edition. *P* values were considered statistically significant if they were less than 0.05. Descriptive statistics were calculated for all applicable variables. For continuous variables with normal distributions we used means and standard deviations, for continuous variables with skewed distributions we used medians and interquartile ranges, and for categorical variables we used frequencies and percentages.

Generalized estimating equation (GEE) models were used to study the effects of the primary predictors (PA level and impact exercise variables; each had their own model) and covariates (BMI and smoking habits) on vertebral CSA (longitudinal outcome). All the analyses were conducted separately for men and women.

First, we conducted both crude and adjusted analyses to study whether PA and impact exercise were associated with vertebral CSA. We also assessed models which included the PA*Time and Impact*Time interaction terms in order to study whether PA and impact exercise affected the vertebral growth rate over the follow-up. We documented the beta coefficients and their 95% confidence intervals from the models.

### 2.6 Ethical considerations

All self-reported data and clinical data was collected with written consent from the individuals and from their parents (when suitable) and the data was treated anonymously. We have followed the Declaration of Helsinki. Ethical Committee of the Northern Ostrobothnia Hospital District in Oulu, Finland, has given us an ethical approval.

## 3 Results

### 3.1 Study sample

The sample consisted of 147 men and 228 women who were on average 21 years old at the baseline MRI and 30 years old at the follow-up (Table 1). More individuals among men belonged to the groups ‘active’ (PA = 4 or more times/week) and ‘high impact’ (1 or more times/week) than among women.

**Table 1:** General characteristics of the sample (*n* = 375).

Characteristic	Men	Women
Sex^a^	147 (39.2)	228 (60.8)
MRI charasteristics		
Age^b^ at baseline MRI (yrs)	21.2 (0.6)	21.3 (0.6)
Age^b^ at follow-up MRI (yrs)	30.5 (0.6)	30.8 (0.5)
MRI interval^b^ (yrs)	9.3 (0.8)	9.4 (0.7)
CSA^b^ at baseline MRI (mm^2^)	1204,91 (154,61)	955,80 (108,43)
CSA^b^ at follow-up MRI (mm^2^)	1291,21 (160,33)	1020,87 (111,48)
Anthropometry, smoking and exercise habits at age 16^c^		
Body height^b^ (cm)	175.3 (7.2)	163.9 (6.0)
Body weight^b^ (kg)	66.0 (12.5)	55.8 (9.1)
Smoking^a^		
Non-smokers	56 (47.9)	77 (42.5)
Occasionally-smokers	20 (17.1)	47 (26.0)
Smokers	41 (35.0)	57 (31.5)
Physical activity^a^		
<1 time/week	22 (15.0)	57 (25.1)
1–4 times/week	72 (49.0)	120 (52.9)
4 or more times/week	53 (36.1)	50 (22.0)
Participation in impact exercise^a^		
Low (<1 time/week)	54 (36.7)	135 (59.5)
High (1 or more times/week)	93 (63.3)	92 (40.5)
Summed Impact^d^	1 (2)	0 (1)

MRI = magnetic resonance imaging, CSA = cross-sectional area. The methodology behind the Summed Impact variable is as explained in Section 2.4.

a n (%).

b Mean (standard deviation).

c N varied due to missing data.

d Median (interquartile range).

We could not detect any statistically significant associations between vertebral CSA, PA, and high-impact exercise in our study sample (Table 2, Table 3, Table 4). The models which contained PA*Time and Impact*Time interaction terms also yielded analogous results.

**Table 2:** Influence of impact exercise on vertebral CSA.

Impact				
	Men	*p*-Value	Women	p-Value
	Beta coefficient (95% CI)		Beta coefficient (95% CI)	
Crude	22.865 (−26.988–72.717)	0.369	13.248 (−15.226–41.722)	0.362
Adjusted^a^	25.566 (−25.025–76.156)	0.322	12.499 (−17.334–42.331)	0.412
Interaction Impact*time	1.108 (−13.653–15.869)	0.883	2.254 (−8.642–13.151)	0.685

CSA = cross-sectional area. These results represent the comparison of low impact (<1 time per week) and high impact (1 or more times per week) exercise. Beta coefficient shows the effect of low participation on the vertebral CSA when comparing it to the effect of high participation, i.e. the beta coefficients stand for the mean difference between observed groups. CI = confidence interval.

a Adjusted for: BMI, smoking.

**Table 3:** Influence of physical activity on vertebral CSA.

Physical activity					
		Men	p-Value	Women	p-Value
		Beta coefficient (95% CI)		Beta coefficient (95% CI)	
Crude	<1 time/week vs. reference	−48.783 (−127.480–29.914)	0.224	23.188 (−14.910–61.286)	0.233
	1–4 times/week vs. reference	−8.119 (−63.583–47.344)	0.774	−3.356 (−33.415–26.703)	0.827
Adjusted^a^	<1 time/week vs. reference	−42.630 (−136.124–50.864)	0.371	2.048 (−37.999–42.094)	0.920
	1–4 times/week vs. reference	−36.271 (−99.243–26.700)	0.259	−9.727 (−44.388–24.934)	0.582
PA*time interaction	(<1 time/week)*time vs. reference	−8.529 (−30.595–13.536)	0.449	−0.942 (−16.013–14.128)	0.902
	(1–4 times/week)*time vs. reference	4.658 (−12.537–21.852)	0.595	6.526 (−6.510–19.563)	0.327

CSA = cross-sectional area, PA = physical activity, CI = confidence interval. Beta coefficient shows the effect of low PA (<1 time per week) on the vertebral CSA and the effect of medium PA (1–4 times per week) when comparing them to the reference, i.e., high PA (4 or more times/week). The beta coefficients stand for the mean difference between observed groups.

a Adjusted for: BMI, smoking.

**Table 4:** Influence of summed value of high-impact sports (continuous score) on vertebral CSA.

Participation in impact exercise				
	Men	p-Value	Women	p-Value
	Beta coefficient (95% CI)		Beta coefficient (95% CI)	
Crude	−1.136 (−18.671–16.398)	0.899	−8.464 (−25.536–8.608)	0.331
Adjusted^a^	−2.229 (−20.343–15.884)	0.809	−14.220 (−33.478–5.038)	0.147
Interaction summed_impact*time	−2.445 (−20.382–15.491)	0.789	−4.438 (−11.337–2.501)	0.210

CSA = cross-sectional area, CI = confidence interval. Beta coefficient shows the effect of one impact point on vertebral CSA.

a Adjusted for: BMI, smoking the methodology behind the Summed Impact variable is as explained in Section 2.4.

## 4 Discussion

This study was conducted in order to find out the associations between the vertebral dimension change and the level of PA as well as the associations between the vertebral CSA and high-impact exercise at early adulthood. We did not find any statistically significant associations between vertebral CSA and PA, or between vertebral CSA and impact sports in either sex. In light of our results, PA in adulthood does not influence the growth rate of the vertebral body. In addition, individuals do not seem to benefit from impact sports either, regarding the growth of vertebral CSA. We concluded that exercising does not help gaining bigger vertebrae and thus prevent vertebral fractures via increased vertebral size.

Our results are thus in line with earlier studies (Junno et al., 2013; Junno et al., 2011; Nikander et al., 2010; Cöster et al., 2016). Several studies suggest that the level of PA or participation in high-impact exercise in early adulthood does not affect the growth of vertebral CSA. However, PA could have some other beneficial effects on L4 (Cöster et al., 2016; Stattin et al., 2017). For example, Tenforde et al. (Tenforde et al., 2018) concluded in their study that participation in high-impact sport increases the BMD among collegiate athletes. Additionally, objectively measured PA was recently shown to be associated with vertebral CSA among both sexes (Modarress-Sadeghi et al., 2019). In addition, PA is known to improve balance and muscle health which as such prevent fallings and fractures (US Department of Health and Human Services, 2008). Conversely, according to a meta-analysis of 22 cohort studies, consisting 1,235,768 individuals and 927 vertebral fractures, the level of PA does not have any effect regarding the risk of vertebral fractures (Qu et al., 2014). Similarly to this study, Nikander et al. (Nikander et al., 2010) found no exercise effect on bone strength.

The main strength of our study was our longitudinal MRI data. Thanks to our two timepoint data, we could observe the associations of PA and high-impact sports with the change of vertebral CSA in the way that is more specific and accurate than previous results from cross-sectional studies. Another strength was our large questionnaire data from several years with wide spectrum of different leisure time sports which allowed us to analyse the association between PA and vertebral dimensions extensively.

In our study, this lack of association can be explained by our relatively small sample size. Another possible reason for this is that the exercise data was collected from self-reported data. Also, as our aim was to investigate how PA in adolescence predicts vertebral size, our activity data was collected at the age of 16–19 and the change in the CSA was measured between ages 20 and 30. It is also possible that there is no association. The wide 95% confidence interval may reflect the small case number. High-impact exercise and summed impact (Table 2, Table 4) seem to have no beneficial effect on the vertebral CSA. The negative beta coefficients in Table 4 even suggest that excessive participation in high-impact sports may have disadvantageous influence in the CSA. Although some of the beta coefficients were negative, suggesting an inverse association between exercise level and vertebral CSA, without statistical significance we cannot assume any reliable association. As our study design clearly differs from previous studies due to longitudinal setting, we believe that our study provides further evidence upon previous results (Nikander et al., 2010; Oura et al., 2017a; Oura et al., 2017c; Qu et al., 2014) with a new perspective. Another limitation in our study was that we did not have data on individuals' BMD and thus we could not study the influence of high-impact sports and PA on the BMD. Although it is possible to obtain MRI derived BMD (Di Iorgi et al., 2018), we used clinical spine imaging protocol (Autio et al., 2019), which did not include suitable sequences for this.

We acknowledge that skeletal maturation and vertebral growth are complex issues with great deal of variation (e.g. population and sex specific). However, we decided to choose the age of 20 years as in most cases epiphyseal closure is almost complete at that timepoint. As we were interested to observe the potential change in vertebral dimensions towards the peak bone mass, a sufficient follow-up interval was required. We chose to observe the vertebral growth at age of 20 and 30 because the bone mass peaks at mid-thirties (Mohammadi et al., 2015). Regarding the best timepoints to provide us sufficient follow-up, we presumed that giving the time of general skeletal maturation at early twenties and of peak bone mass would provide the most favourable outcome. We chose to observe high-impact sports because of their beneficial character on BMD and bone geometry (Tenforde and Fredericson, 2011). Gymnastics was excluded from high-impact sports because in Finnish the term ‘gymnastics’ (‘voimistelu’) is nonspecific. PA in young adulthood has been shown to predict PA in later adulthood (Engström, 1986; Barnekow-Bergkvist et al., 1998), and this has been observed also among Northern Finns (Tammelin, 2003). The observed smoking rate in our study sample was similar to the general Finnish population (Jääskeläinen and Virtanen, 2019).

In general, childhood is also a very interesting time regarding the influence of PA because in children the bone geometry changes in response to PA (Heaney et al., 2000). This raises the question, whether there is one or several dominant timepoints in childhood or in early adulthood where we can try to modify the vertebral dimensions (for example with PA or nutrition) in a way that individuals could have a lifelong advantage and reduced fracture risk.

In conclusion, neither physical activity nor high-impact sports seem to be associated with the change in vertebral CSA among young adults. Future studies should provide new insights into managing the risks of vertebral fractures. Because besides just vertebral dimensions, also low BMD is associated with higher fracture risk, future studies should include information on both bone geometry and BMD.

## CRediT authorship contribution statement

**Elsi Autio:** Writing – original draft, Formal analysis, Conceptualization. **Petteri Oura:** Writing – original draft, Visualization, Formal analysis, Validation, Conceptualization. **Jaro Karppinen:** Writing – review & editing. **Markus Paananen:** Writing – review & editing. **Juho-Antti Junno:** Writing – original draft, Project administration, Validation, Conceptualization. **Jaakko Niinimäki:** Supervision, Writing – review & editing.

## Declaration of competing interest

The authors have no conflict of interest.
